# GDNF secreted from adipose-derived stem cells stimulates VEGF-independent angiogenesis

**DOI:** 10.18632/oncotarget.9208

**Published:** 2016-05-06

**Authors:** Zhaohui Zhong, Huiying Gu, Jirun Peng, Wenzheng Wang, Brian H. Johnstone, Keith L. March, Martin R. Farlow, Yansheng Du

**Affiliations:** ^1^ Department of General Surgery, Peking University People's Hospital, Beijing 100044, PR China; ^2^ Department of Neurology, Indiana University School of Medicine, Indianapolis, IN 46202, USA; ^3^ Department of Surgery, Beijing Shijitan Hospital, Capital Medical University, Beijing 100038, PR China; ^4^ Ninth Clinical Medical College of Peking University, Beijing 100038, PR China; ^5^ Department of Medicine, Indiana University School of Medicine, Indianapolis, IN 46202, USA; ^6^ Indiana Center for Vascular Biology and Medicine, Indiana University School of Medicine, Indianapolis, IN 46202, USA; ^7^ Krannert Institute of Cardiology, Indianapolis, IN 46202, USA; ^8^ VA Center for Regenerative Medicine, Indina University School of Medicine, Indianapolis, IN 46202, USA; ^9^ School of Health Sciences, Purdue University, West Lafayette, IN 47907, USA

**Keywords:** ASC-CM, GDNF, VEGF, angiogenesis, hepatocellular carcinoma

## Abstract

Adipose tissue stroma contains a population of mesenchymal stem cells (MSC) promote new blood vessel formation and stabilization. These adipose-derived stem cells (ASC) promote de novo formation of vascular structures in vitro. We investigated the angiogenic factors secreted by ASC and discovered that glial-derived neurotrophic factor (GDNF) is a key mediator for endothelial cell network formation. It was found that both GDNF alone or present in ASC-conditioned medium (ASC-CM) stimulated capillary network formation by using human umbilical vein endothelial cells (HUVECs) and such an effect was totally independent of vascular endothelial growth factor (VEGF) activity. Additionally, we showed stimulation of capillary network formation by GDNF, but not VEGF, could be blocked by the Ret (rearranged during transfection) receptor antagonist RPI-1, a GDNF signaling inhibitor. Furthermore, GDNF were found to be overexpressed in cancer cells that were resistant to the anti-angiogenic treatment using the VEGF antibody. Cancer cells in the liver hepatocellular carcinoma (HCC), a non-nervous related cancer, highly overexpressed GDNF as compared to normal liver cells. Our data strongly suggest that, in addition to VEGF, GDNF secreted by ASC and HCC cells, may be another important factor promoting pathological neovascularization. Thus, GDNF may be a potential therapeutic target for HCC and obesity treatments.

## INTRODUCTION

Angiogenesis is responsible for most, if not all, blood vessel growth during development and disease pathogenesis [1]. This process could be a target for combating diseases characterized by either poor vascularization or abnormal vasculature. Some diseases, such as ischemic chronic wounds in brain and heart, are the result of failure or insufficient blood supply and could be treated by a local expansion/formation of blood vessels, thus bringing new nutrients to the site, facilitating repair [2]. In contrast, other diseases like obesity and solid tumors, angiogenesis is required for their growth and metastasis by supplying nutrients, oxygen, and removing wastes as well as transporting cancer cells to a distant site [3, 4]. Tumor vessels also have the theoretical potential for developing acquired resistance to drugs to escape therapy [5]. Therefore, angiogenesis inhibition prevents the formation of new blood vessels, thereby stopping or slowing the expansion of adipose tissue in obesity as well as the growth or spread of tumors.

Mesenchymal stem cells (MSC) isolated from many different tissues all secrete potent proangiogenic factors [6]. Adipose-derived stem cells (ASC) are pluripotent MSC in the stromal or non-adipocyte fraction of the adipose tissue and were demonstrated to exhibit differentiation into endothelial as well as smooth muscle-like cells and induce new neovascularization of ischemic tissues [7–10]. Recently, it was reported that recruitment of endogenous ASC from fat tissue is particularly associated with increased vascularization and adipogenesis accompanied by proliferation of malignant cells [11]. Like cancer cells [12–14], ASC secret many angiogenic factors including VEGF, hepatocyte growth factor (HGF) and fibroblast growth factor (FGF), which stimulate neovascularization in vivo and vascular network formation in vitro [6, 10] and widely used to establish an in vitro angiogenic model [10]. Generally speaking, many growth factors involved in angiogenesis, including VEGF, PDGF, and FGF, are secreted by ASC [6, 10, 15, 16] and cencer cells [12–14, 17, 18]. VEGF is well accepted as the major angiogenic factor for both stem cells and cancer cells, and three classes of agents that mainly target VEGF have been developed in research or clinical practice: monoclonal antibodies, VEGF decoy receptors, and small molecule tyrosine kinase inhibitors (TKIs). Anti-angiogenic agents are currently used in the monotherapy or in combination with cytotoxic chemotherapy or radiation for treating cancers [19] and also proposed for obesity treatment [20]. However, despite much effort, major concerns remain in using the antiangiogenic approach for cancer treatment, including tumor resistance due to overcompensation from the parallel growth factors or signaling pathways. In the obesity treatment, although several angiogenesis inhibitors could markedly reduce body weight [21], inhibition of VEGF-dependent angiogenesis might not play an important role in these cases since it had been shown that anti-VEGF treatment did not induce significant weight loss in mice [22]. Further research is therefore necessary to improve the existing treatments by combinations of these agents simultaneously targeting multiple anti-angiogenic pathways for cancer and obesity treatments.

Glial cell line-derived neurotrophic factor (GDNF) is a member of the transforming growth factor-beta superfamily and possesses potent neuroprotective effect on a variety of neuronal damage [23, 24]. Topical application and intracerebral administration of GDNF significantly decreased the size of ischemia-induced brain infarction and the number of TUNEL-positive neurons with suppression of the apoptotic pathways [25]. Additionally, it was also demonstrated that GDNF might promote vessel integrity in cultured neonatal rabbit explants containing the nephrogenic zone of the kidney [26] and assumed GDNF was possibly involved in angiogenesis through the VEGF pathway [26]. However, the actual mechanism by which GDNF and the RET receptor (the rearranged during transfection (RET) proto-oncogene product, a tyrosine kinase receptor) that is involved in GDNF signaling induce angiogenesis and whether this process involves VEGF has not been determined. The mRNA for GDNF was recently detected in ASC by RT-PCR; however, it was not determined if these cells express functional GDNF protein [27]. In this study we demonstrate that active GDNF protein is indeed expressed by ASC in culture and potently promotes angiogenesis in vitro. Since ASC potentially promote carcinogenesis by homing to tumors, where they promote angiogenesis [11, 28, 29], we investigated whether and how GDNF contributes to new blood vessel formation, either alone or in combination with VEGF. Additionally, since angiogenesis in cancer development may be stimulated by both adipose and cancer cells in a similar way [30], we further investigated whether GDNF could also be expressed and secreted by liver hepatocellular carcinoma (HCC) cells that are non-nervous and not response to anti-VEGF treatments. In this study, we discovered that GDNF was present in HCC but not in surrounding normal liver tissues. Together, the findings of GDNF expression by ASC and HCC cells suggest new potential therapeutic targets for cancer and obesity treatments.

## RESULTS

### ASC-CM markedly stimulated the capillary-like tube formation of HUVEC in matrigel and VEGF in the ASC-CM played a significant role in this process

**Figure 1:**
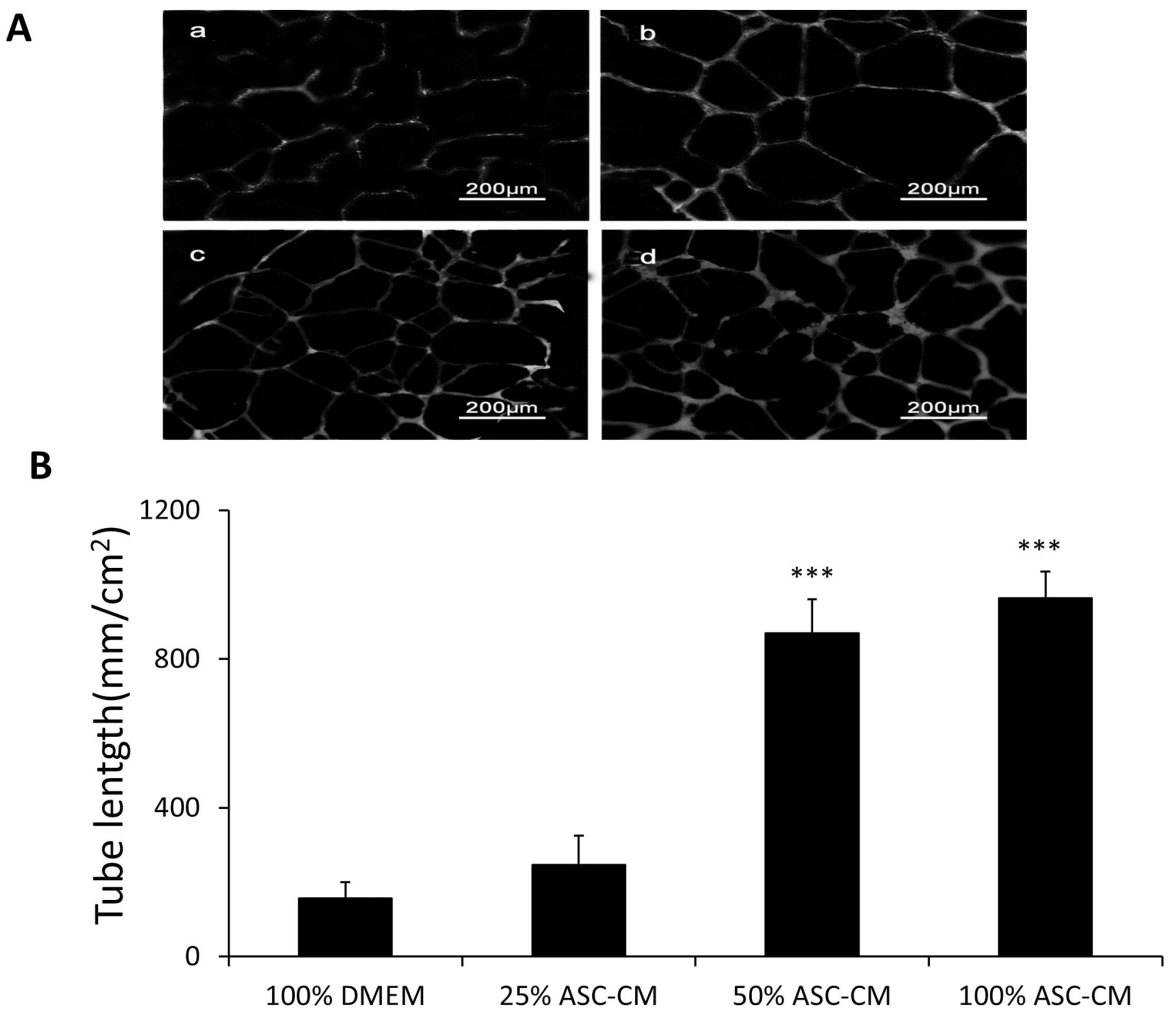
ASC-CM stimulated capillary network formation by HUVEC in Matrigel HUVECs suspended in Matrigel were subjected to different doses of ASC-CM treatments or DMEM and analyzed for capillary network formation after 48 hours. **A.** Representative images demonstrating capillary network formation of HUVECs in Matrigel media. a: 100% DMEM; b: 25% ASC-CM in DMEM; c: 50%ASC-CM in DMEM; d: 100 % ASC-CM. **B.** Capillary networks were stained and analyzed as described in Methods. Data of tube length represent mean ± SD of three independent experiments. N=3, *** p<0.001 compare with 100% DMEM.

To investigate the angiogenic effects of ASC-CM on tube formation by endothelial cells, we conducted in vitro experiments using HUVECs which form tube-like structure after 48 hours cultures in Matrigel. ASC-CM stimulated tube formation in a dose-dependent manner (DMEM: 157.2 ± 50.5 mm/cm^2^, 25% ASC-CM: 246.8 ± 77.5 mm/cm^2^, 50% ASC-CM: 869.8 ± 90.4 mm/cm^2^, 100% ASC-CM: 964.1 ± 71 mm/cm^2^, p< 0.001) (Figure 1). Since previous evidence showed that VEGF was a key player in the tube formation, we examined how much VEGF in the ASC-CM was involved in ASC-CM-stimulated tube formation. We measured the VEGF level in the ASC-CM by using ELISA and detected a significant level of VEGF (0.91 ± 0.05 ng/ml) that was present in the ASC-CM. As expected, capillary network formation was diminished by 60% through inactivation of VEGF in the ASC-CM (686 ± 107 mm/cm^2^ vs. 263 ± 71 mm/cm^2^, p<0.001) (Figure 4).

### GDNF in the ASC-CM also promoted capillary network formation at a similar degree to VEGF did

**Figure 2:**
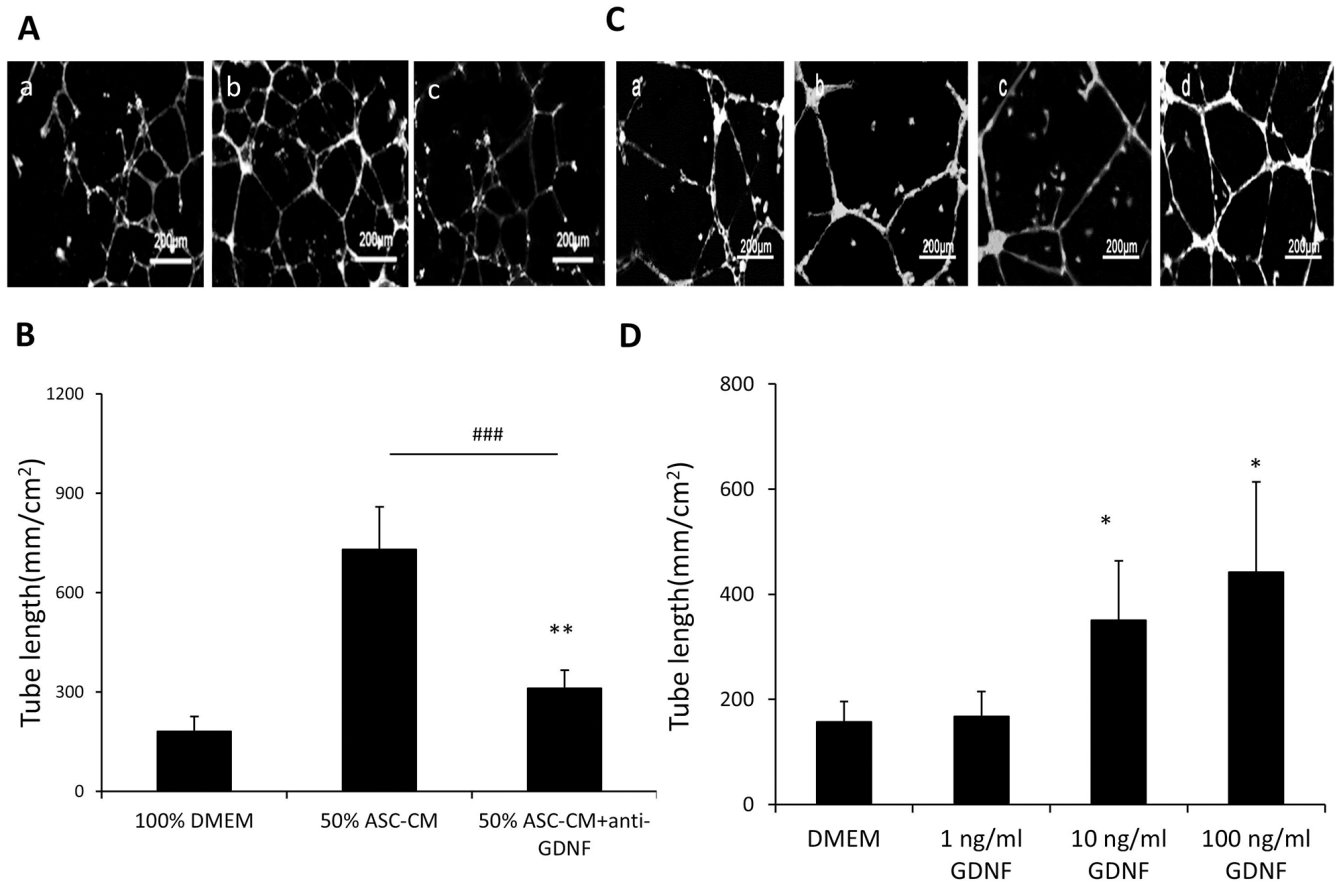
GDNF in ASC-CM and recombinant protein stimulated the network formation of HUVECs in Matrigel **A.** GDNF antibody inhibited ASC-CM-stimulated capillary network formation. Representative images demonstrating the tube network formation of HUVECs in Metrigel. a: 100% DMEM; b: 50% ASC-CM c: 50% ASC-CM+GDNF antibody. The tube length in three wells of each treatment was compared to those without ASC-CM treatments (total length ± SD). **: p<0.01, ***: p<0.001, compared with the group of 100% DMEM. **B.** GDNF stimulated the network formation of HUVECs in Matrigel. Representative images demonstrating the tube network formation of HUVECs in Matrigel. a: DMEM; b: DMEM+ 1ng/ml GDNF; c: DMEM+ 10ng/ml GDNF; d: DMEM+ 100ng/ml GDNF. Analysis was performed at 48 hours following ASC-CM treatments. Data of tube length represent mean ± SD of three independent experiments. N=3, *: p<0.05, compared with the group of DMEM.

**Figure 3:**
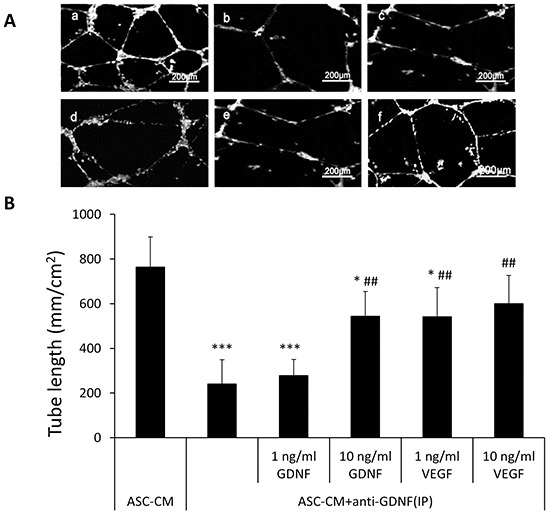
Addition of GDNF or VEGF back to ASC-CM depleted of GDNF was able to recover ASC-CM-induced tube network formation of HUVECs **A.** Representative images demonstrating the tube network formation of HUVECs in Metrigel. a: 50% ASC-CM; b: 50% ASC-CM+ anti-GDNF (IP); c: 50% ASC-CM+ anti-GDNF (IP)+ 1ng/ml GDNF; d: 50% ASC-CM+ anti-GDNF (IP)+ 10ng/ml GDNF; e: 50% ASC-CM+ anti-GDNF (IP)+ 1ng/ml VEGF; f: 50% ASC-CM+ anti-GDNF (IP)+ 10ng/ml VEGF. **B.** Analysis was performed at 48 hours following ASC-CM treatments. Data of tube length represent mean ± SD of three independent experiments. N=3, *: p<0.05, ***: p<0.001, compared with the group of ASC-CM, ##: p<0.01, compared with the group of ASC-CM+ anti-GDNF (IP).

In addition to the many angiogenic factors previously shown to be produced by ASC, these cells also secreted a significant level of GDNF (23.6 ±1.35 ng/ml) that was detected by ELISA. Neutralization of GDNF using an inactivating antibody abolished the effect of ASC-CM on endothelial network formation at a similar degree as inactivation of VEGF did (764 ± 134 mm/cm^2^ vs. 240 ± 108 mm/cm^2^, p<0.01, Figure 2) (Figure 2A). Additionally, addition of recombinant GDNF individually promoted capillary network formation in a dose fashion (0: 157 ± 38 mm/cm^2^, 1ng/ml GDNF: 168 ± 38 mm/cm^2^, 10ng/ml GDNF: 351 ± 112 mm/cm^2^, 100ng/ml GDNF: 442 ± 171 mm/cm^2^) (Figure 2B). For further confirmation, 10 ng/ml of recombinant GDNF was added to ASC-CM that endogenous GDNF had been depleted by using immunoprecipitation with an anti-GDNF antibody and the mixture dramatically recovered the tube formation activity (240 ± 108 mm/cm^2^ vs. 544.43 ± 110 mm/cm^2^, p< 0.01) (Figure 3). Interestingly, addition of exogenous VEGF at either 1 or 10 ng to GDNF-depleted ASC-CM also re-established network formation activity (240 ± 108 mm/cm^2^ vs. 542 ± 130 mm/cm^2^ or 601 ± 125 mm/cm^2^, p< 0.01) (Figure 3), indicating levels of endogenous VEGF in GDNF-depleted ASC-CM was not enough to stimulate angiogenesis, but could be helped by addition of either exogenous GDNF or VEGF.

### Capillary network formation stimulated by GDNF was independent of VEGF activity

**Figure 4:**
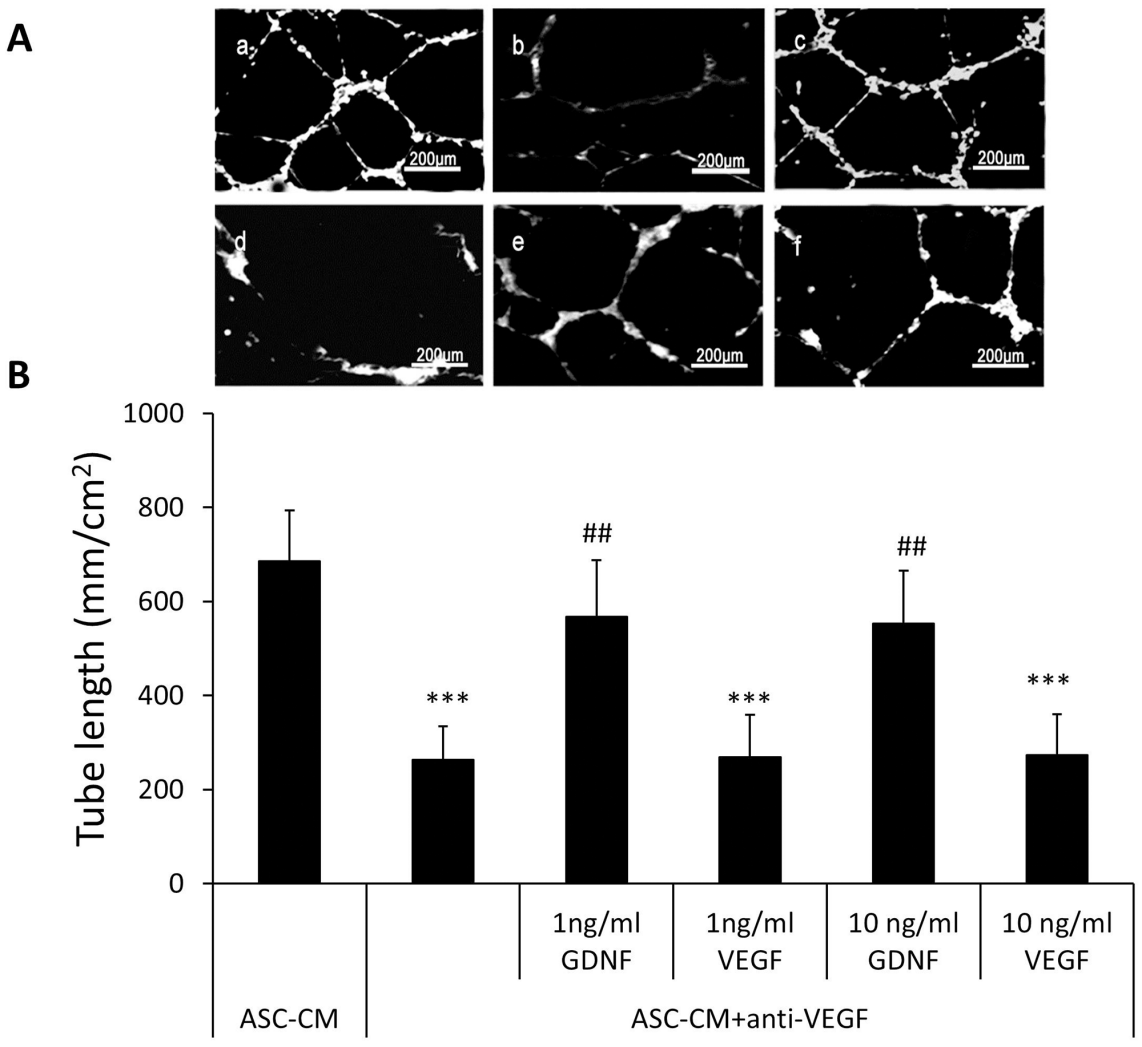
Addition of GDNF into ASC-CM pretreated with the excess amount of the VEGF antibody was able to stimulate HUVECs tube formation **A.** Representative images demonstrating the tube network formation of HUVECs in Metrigel. a: 50% ASC-CM; b: 50% ASC-CM+ anti-VEGF; c: 50%ASC-CM+ anti-VEGF+ 1 ng/ml GDNF; d: 50% ASC-CM+ anti-VEGF+ 10 ng/ml GDNF; e: 50% ASC-CM+ anti-VEGF+ 1 ng/ml VEGF ; f: 50% ASC-CM + anti-VEGF+ 10 ng/ml VEGF. **B.** Analysis was performed at 48 hours following ASC-CM treatments. Data of tube length represent mean ± SD of three independent experiments. N=3, ***: p<0.001, compared with the group of ASC-CM; ##: p<0.01, compared with the group of ASC-CM+ anti-VEGF.

**Figure 5:**
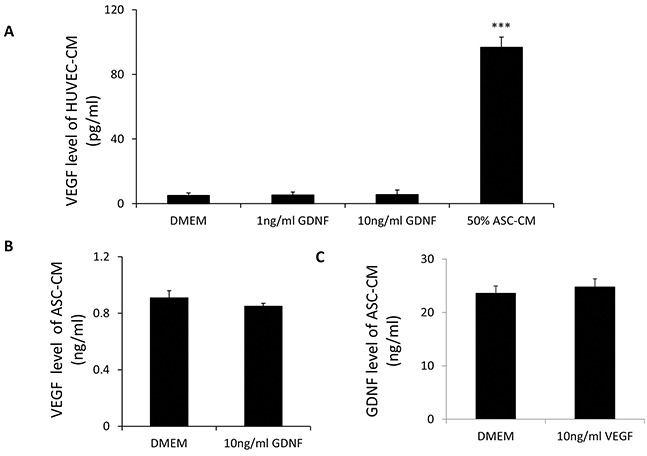
**A, B.** GDNF treatments did not stimulate VEGF secretion in HUVECs and ASC. 24-hour treatments of GDNF at 10 ng/ml did not affect VEGF production in HUVECs and ASC. **C.** VEGF did not affect GDNF production in ASC. 10 ng/ml VEGF treatments did not affect GDNF production in ASC. Data represent mean ± SD of three independent experiments. N=3, ***: p<0.001, compared with the group of DMEM.

Since a previous study suggested that GDNF may induce angiogenesis through the VEGF pathway, we investigated whether GDNF was able to induce angiogenesis in the absence of VEGF. We pre-incubated 1 ml ASC-CM with 2 ml VEGF neutralizing antibody at the concentration of 500 mg/ml (Abcam, Cambridge, MA) for 30 min to remove endogenous VEGF activity and demonstrated that exogenous VEGF was not able to induce capillary network formation in this media (263 ± 71 mm/cm^2^ vs. 269 ± 89 mm/cm^2^ or 274 ± 85 mm/cm^2^) by adding external VEGF (1 or 10 ng/ml) into this VEGF-neutralized ASC-CM. Interestingly, addition of recombinant GDNF into this VEGF-neutralized ASC-CM even at as low as 1 ng/ml still significantly stimulated capillary network formation (263 ± 71 mm/cm^2^ vs. 568 ± 119 mm/cm^2^, P<0.01) (Figure 4). It should be noted that in Figure 3, we used the immunoprecipitation method and the anti-GDNF antibody was removed before adding ASC-CM. In contrast, in Figure 4, we used the VEGF neutralizing antibodies to neutralize the VEGF. The addition of GDNF at 1 ng/ml into ASC-CM pretreated with the excess amount of VEGF antibody was able to stimulate HUVECs tube formation, indicating that GDNF was able to exert the angiogenic function without the presence of VEGF activity. For further confirmation, we showed that treatment of HUVEC with 10 ng/ml GDNF did not stimulate VEGF secretion (Figure 5A, 4.98 ± 1.54 pg/ml VS. 5.49 ± 2.92 pg/ml). Additionally treatment of ASC with 10 ng/ml GDNF did not affect VEGF secretion (Figure 5B, 0.91 ± 0.05 ng/ml vs. 0.85 ± 0.04 ng/ml) and VEGF did not modulates GDNF expression in ASC (23.6 ±1.35 ng/ml vs. 2.48 ± 1.52 ng/ml).

### Inhibition of RET receptor, a downstream factor of the GDNF signaling pathway blocked GDNF–induced capillary network formation

**Figure 6:**
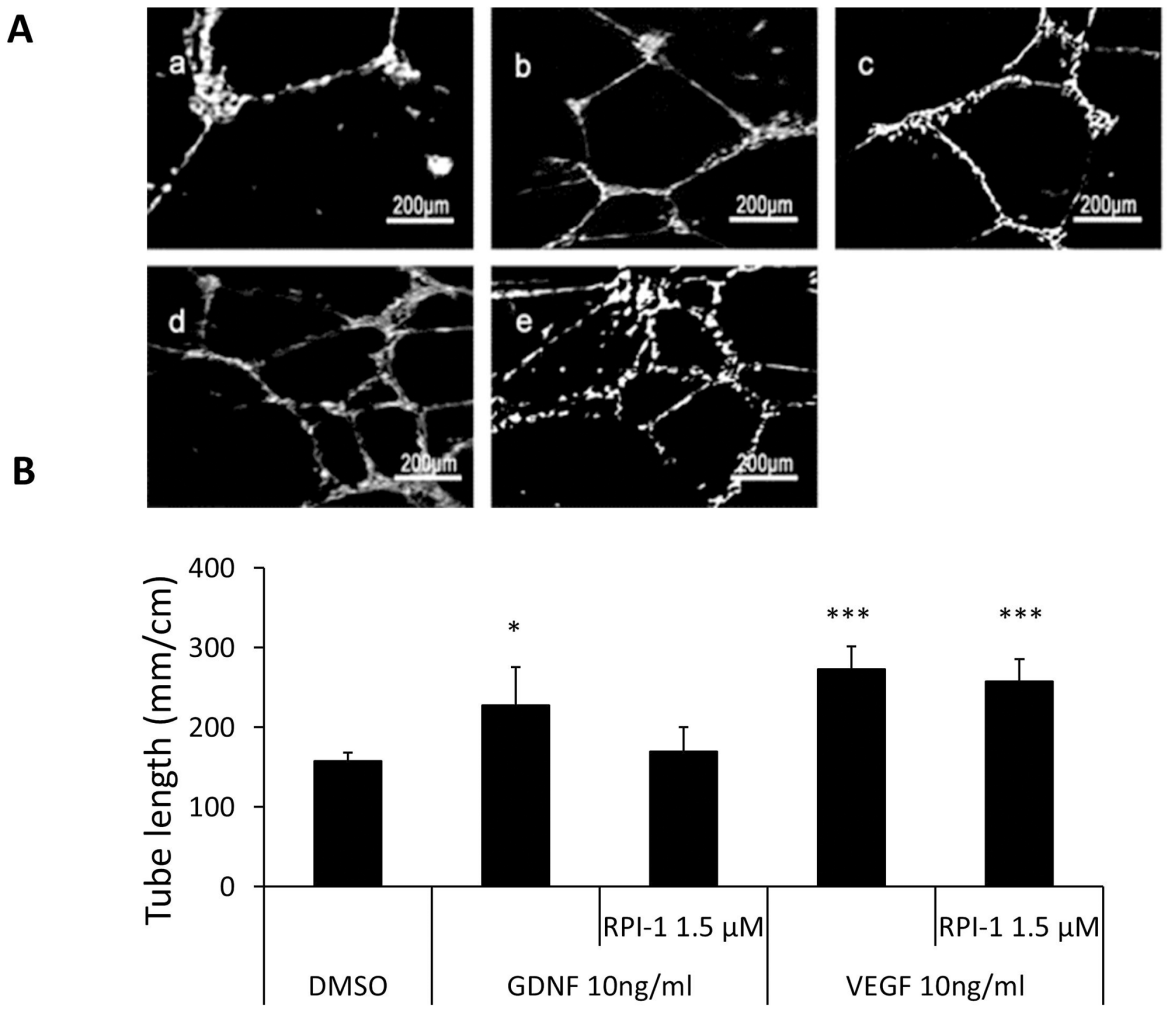
RPI-1, the inhibitor of the GDNF receptor (RET), affects GDNF, but not VEGF, induced HUVECs tube formation **A.** Representative images demonstrating the tube network formation of HUVECs in Matrigel. a: control: DMSO; b: 10 ng/ml GDNF; c: 10 ng/ml GDNF+ 1.5 μM RPI-1; d: DMEM+ 10ng/ml VEGF; e: DMEM+ 10ng/ml VEGF+ 1.5 μM RPI-1. **B.** Analysis was performed at 48 hours following ASC-CM treatments. Data of tube length represent mean ± SD of three independent experiments. N=3, *: p<0.05, ***: p<0.001, compared with the control group of DMSO.

We used RPI-1 to further confirm GDNF directly induced capillary network formation. RPI-1 is a competitive inhibitor of the ATP-dependent RET kinase receptor that is involved in GDNF signaling [31, 32]. As expected, addition of the RPI-1 potently blocked capillary network formation induced by GDNF, indicating that activation of the RET co-receptor was required for the angiogenic properties of GDNF (Figure 6). In contrast, RPI-1 did not affect VEGF-induced capillary network formation.

### GDNF is overexpressed in human HCC

**Figure 7:**
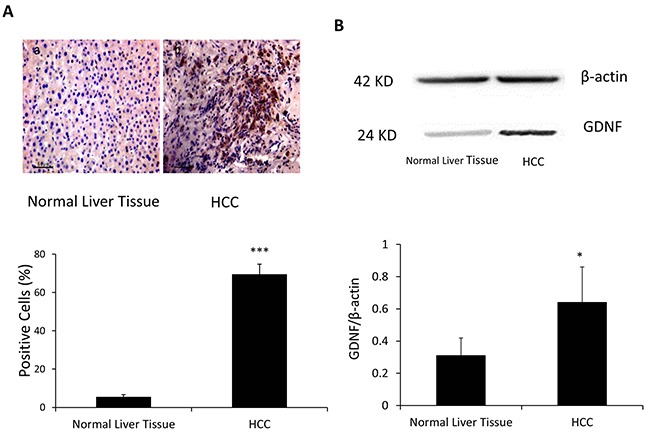
GDNF was overexpressed in the HCC as compared to the normal liver tissue **A.** Immunohistological studies by using GDNF antibodies were performed in the HCC and normal liver tissue. a: normal liver tissue b: HCC. **B.** Western blot analysis of GDNF expression in normal liver tissue and HCC. Band intensities by western blot were quantified by normalizing to β-actin. N=42, *: p<0.05, ***: p<0.001, compared with control group of normal liver tissue.

At last, we investigated whether GDNF, as an independent angiogenic factor, was also secreted by HCC cells since Bevacizumab, the VEGF antibody could not be used to effectively treat HCC [33]. An immunohistological analysis of human HCC specimens was performed by using the anti-GDNF antibody and our results revealed in 42 patients, there were dramatically increased expression levels of the GDNF in HCC, as compared to normal liver tissue from same patient in all tested specimens (Figure 7A, 69.4 ± 5.4% VS 5.3 ± 1.3%, p<0.001). The Western blot was also used to confirm that the expression of GDNF in HCC tissue was significantly higher than normal liver tissue (0.64±0.22 VS 0.31±0.11, p<0.05) (Figure 7B). This finding further supports that even blockade of VEGF angiogenic function, highly expressed GDNF in HCC cells may continuously stimulate HCC angiogenesis and progression.

## DISCUSSION

ASC have been previously demonstrated to be involved in angiogenesis and vascular formation through direct contact with endothelial cells and by provision of angiogenic growth factors, such as VEGF, HGF, platelet-derived growth factor (PDGF) and metalloproteinase [10, 15]. Each of these factors was shown to be important for de novo vessel formation in vitro [15]. However, ASC secrete many other factors with demonstrated or potential pro-angiogenic activities; therefore, it is possible that other factors present in ASC-CM could influence vessel growth, especially under pathophysiological conditions. Certainly, FGF that is key factor for stem cell survival has been considered as a major factor in cancer angiogenesis [34].

ASC has recently been widely investigated in angiogenesis because it can be used to treat brain and heart ischemia partially through recovering blood supply into ischemic areas [15, 35, 36]. Additionally, fat tissues, particularly white fat tissues, in addition to involving in obesity development, were found to be involved in carcinogenesis and related angiogenesis [34]. Factors secreted from ASC were shown to be definitely involved in building a microenvironment that enhanced angiogenesis in cancer [37–39]. Therefore, understanding the function of those factors that play key role in angiogenesis is necessary for effective obesity, cancer and ischemic injury treatment.

Blocking angiogenesis in cancer development is an attractive therapeutic treatment not only because it could be used to treat a wide spectrum of cancers, but also provides a new class of treatments with low-toxicity/side effects [40]. Bevacizumab, however, a monoclonal antibody targeting VEGF used clinically to treat cancers has a limited number of cancer targets and low efficacy, which limits its range of usefulness. Antiangiogenic therapy for cancer has met with a number of hurdles on its way to becoming an option for cancer therapy [41–43]. Multiple clinic trial have demonstrated that the benefit of VEGF-targeted therapy is variable and depends, in part, on the tumor type, stage and treatment history [44]. The primary concern is whether other targets that promote cancer angiogenesis can compensate for VEGF depletion. Reports indicated that FGF might induce angiogenesis and its activity must also be neutralized to fully treat cancer [38, 45]. However, the microenvironment for angiogenesis is complicated and may still require identifying and targeting other factors that may independently induce cancer angiogenesis. Recently, angiogenesis inhibitors have also been suggested for obesity treatments since angiogenesis in obesity provides new blood vessels are able to bring nutrients and oxygen to expand fat tissue [20]. However, angiogenesis in obesity is through a complex mechanism since anti-VEGF treatment failed to induce significant weight loss [22]. Here, we used ASC as an investigative model since they have been demonstrated to contribute to angiogenesis in both cancer and ischemic tissues. By using conditioned media comprised of factors secreted by these cells, we clearly demonstrated that both VEGF and GDNF played significant roles in ASC-CM-induced angiogenesis. Interestingly, GDNF, which is usually assumed to function as a neuroprotective factor, was able to induce angiogenesis independently from the VEGF pathway. Ret-1, a specific GDNF receptor, specifically mediates GDNF- but not VEGF-induced angiogenesis. Additionally, GDNF did not affect VEGF expression and, furthermore, blocking VEGF activity did not inhibit GDNF-induced angiogenesis. All of these data strongly suggest that GDNF induces new vessel growth independently of the VEGF angiogenesis pathway. Interestingly, Ret was reported to mediate neurturin (NRTN)-, a member of the GDNF family of ligands (GFL), induced phosphorylation of the signal transducers and activators of transcription 3 (STAT3) [46]. STAT3 played a critical role in oncogenesis and angiogenesis [47, 48]. Therefore, STAT3 signaling is possibly involved in GDNF-induced angiogenesis. Additionally, GDNF, was strongly overexpressed in human liver cancer cells, indicating solely blocking VEGF in these cancers would not be effective in preventing cancer progression and metastasis. Therefore, it should be very interesting to investigate how GDNF is involved in cancer and obesity pathogenesis.

Since GDNF is clearly another independent key player in angiogenesis, induced by adipose stem cells and cancer cells, it is necessary to further investigate its downstream pathways as this factor may closely be involved in angiogenesis in cancer, obesity, and ischemia injury. It should be noted that high doses of VEGF appeared to replace GDNF angiogenic activity when GDNF had been neutralized in ASC-CM, indicating without GDNF, VEGF could also independently induce angiogenesis. The combination of inhibiting both factors would be required for effective therapy on angiogenesis.

## MATERIALS AND METHODS

### Isolation and culture of human ASC

Human subcutaneous adipose tissue samples, obtained from elective lipoaspiration/liposuction procedures were minced before digesting in 1 mg/ml Collagenase Type I solution (Worthington Biochemical Corporation, Lakewood, NJ) under gentle agitation for 1 hour at 37°C. The digested mixture was diluted with 50 ml culture medium (DMEM, high glucose, 10% FBS) and centrifuged at 200g for 5 minutes to separate the stromal cell fraction (pellet) from adipocytes. The supernatant was removed and the cellular pellet was re-suspended in 20 ml of fresh medium (DMEM/10% FBS), and then serially filtered through 250μm Nitex 03-250/50 cloth (Sefar American Inc., Depew, NY) and a 100μm cell strainer (BD Biosciences, Franklin Lakes, NJ) to remove debris. The filtrate was again centrifuged at 200 g for 5 minutes. The pellet containing ASC was treated with red blood cell lysis buffer for 5 minutes at 37°C and then pelleted at 300g for 5 minutes. The cell pellets were re-suspended in the EGM-2MV medium (Lonza, Allendale, NJ). ASC were plated in an uncoated T75 tissue culture flask at a density of 4 × 10^6^ cells/cm^2^ and incubated in a humidified chamber at 37°C in an atmosphere of 5% CO_2_. After overnight culture, the medium was replaced with Endothelial Growth Medium 2-MicroVascular (EGM2-MV, Lonza, Allendale, NJ). The Indiana University ethics committee approved the experiments and all informed consent was obtained from all subjects. All methods were performed in accordance with approved guidelines.

### Collection of ASC conditioned media (ASC-CM) and human umbilical vein endothelial cells conditioned media (HUVEC-CM)

Human ASC were cultured to confluence in 100mm culture dishes containing EGM2-MV medium, and each dish was rinsed and replenished with 5 ml Dulbecco's Modified Eagle's Medium (DMEM, Invitrogen, Grand Island, NY) for 24 hours. Media were collected and centrifuged at 3000 g and stored at −80°C until use. Conditioned medium from mature human umbilical vein endothelial cells (HUVEC-CM) was obtained in parallel and applying the same protocol, respectively. Growth factor-free DMEM with 1% FBS was used as control medium in all experiments.

### Quantification of GDNF and VEGF by ELISA

Levels of GDNF and VEGF in ASC-CM and HUVEC-CM were measured using an enzyme-linked immunosorbent assay (ELISA) kit, Human VEGF Quantikine Elisa kits (R&D system, Minneapolis, MN), according to the manufacturer's instructions [49]. All samples and standards were measured in duplicate.

### Depletion GDNF by immunoprecipitation (IP)

1 ml ASC-CM was incubated overnight at 4°C with 2 μl anti-GDNF antibody (500μg/ml) (Abcam, Cambridge, MA). Protein A-agarose was added, and a second overnight incubation was performed, followed by centrifuging.

### Matrigel assay

Ninety six–well plates were coated with 100 μl per well of growth factor-reduced Matrigel (BD Biosciences, Franklin Lakes, NJ). Plates were incubated overnight at 4°C then at 37°C for 2 hours. HUVECs at passage 6 (10^4^ cells/well) in 100 μl of different medium and were cultured at 37°C with 5% CO_2_ for 1hour, after HUVECs touched the Matrigel, then changed different medium, and monitored after 48hours, took pictures by microscopy. Data analyzed by MetaMorph7.0 IMAGE Analysis Software [50].

### Immunocytochemistry of GDNF in HCC and liver cirrhosis tissue

We performed immunohistochemistry for GDNF in 42 cases of HCC and liver cirrhosis tissue (From Peking University People's Hospital General Surgery Department, Beijing, China). The Peking University People's Hospital ethics committee approved the experiments and all informed consent was obtained from all subjects. All methods were performed in accordance with approved guidelines. After fixation with formaldehyde and endogenous peroxidase activity quenching, sections were incubated with bovine serum albumin for 1 hour, then, they were incubated with primary antibody for GDNF (1:500; Abcam, Cambridge, MA) overnight at 4°C. On the next day, we treated the sections with mouse monoclonal biotinylated secondary antibody (1:200; Cell Signaling, Danvers, MA) for 2 hours at room temperature. Imumuoreactivities were developed in horseadish peroxidase-streptavidin-biotin complex solution (Vectastatin ABC kit; Vector Labs, Burlingame, CA) for 30 minutes and then incubated with diaminobenzidine (DAB; Sigma-Aldrich, St. Louis, MO). Negative controls were included by omitting the primary antibody. The positively stained cells were identified by their morphology, and the staining intensity was measured by Image pro 6 [51]. GDNF was evaluated in the zone of GDNF- positive areas in five sections per HCC or liver tissue.

### Quantitative western blot assay

The protein extract of HCC tissues separated by SDS-PAGE were transferred to PVDF membrane. The blots were probed with GDNF antibody (1:500, Abcam, Cambridge, MA, USA), followed by a secondary antibody conjugated with horseradish peroxidase (1:5000) and visualized by utilizing enhanced chemiluminescence. β-actin was also assayed as loading controls by using its antibody (1:1000). Band intensities were quantified and results were reported as a ratio of GDNF to β-actin [52].

### Statistical analysis

Unless indicated, otherwise, data are given as means ± SD; with the number of determinations (n) representing separate experiments carried out independently using triplicate samples. Data were evaluated using one-way ANOVA and a P value of less than 0.05 was considered significant.

## CONCLUSION

GDNF alone or present in ASC-CM stimulated capillary network formation by HUVECs and this effect was totally independent of VEGF activity. Additionally, the stimulation of capillary network formation by GDNF could be blocked by the GDNF Ret receptor antagonist RPI-1. We also revealed that GDNF was overexpressed in HCC cells that were resistant to the anti-angiogenic treatment using VEGF antibody. Our data strongly suggest that, in addition to VEGF, GDNF secreted by ASC, and possibly non-nervous tumors such as HCC cells, is another important factor promoting pathological neovascularization. Thus, GDNF is a potential therapeutic target for cancer and obesity.
